# ASPM promotes the progression of ovarian endometriosis by modulating the cell cycle and activating the Wnt/β-catenin signaling pathway

**DOI:** 10.1097/MD.0000000000048765

**Published:** 2026-05-15

**Authors:** Xin Liu, Rongyan Qin, Fengque Zheng

**Affiliations:** a Department of Laboratory Medicine, Key Laboratory of Precision Medicine for Viral Diseases, Guangxi Health Commission Key Laboratory of Clinical Biotechnology, Liuzhou People’s Hospital, Liuzhou, Guangxi, China; b Department of Reproductive Medicine, The First Affiliated Hospital of Guangxi Medical University, Nanning, Guangxi, China; c Department of Gynecology, The Fourth Affiliated Hospital of Guangxi Medical University, Liuzhou, Guangxi, China.

**Keywords:** ASPM, endometriosis, invasion, migration, proliferation

## Abstract

**Background:**

Endometriosis (EMs) is a common gynecological disorder associated with impaired fertility and reduced quality of life. This study investigated abnormal spindle-like microcephaly-associated protein (ASPM), identified as a hub gene in EMs pathogenesis, and explored its functional role and molecular mechanisms.

**Materials and methods:**

Bioinformatics analysis identified key genes associated with EMs. ASPM expression was compared between controls and endometrial tissues or primary endometrial stromal cells from EMs patients. In vitro experiments assessed ASPM’s effects on proliferation, invasion, and migration. Transcriptome sequencing revealed ASPM’s downstream signaling pathways. Subsequent in vitro experiments demonstrated that ASPM promotes EMs progression via cell cycle regulation and Wnt/β-catenin signaling.

**Results:**

Our bioinformatics analysis identified ASPM as a key differentially expressed hub gene in EMs. Reverse transcription quantitative polymerase chain reaction, immunohistochemistry, and western blot analyses demonstrated elevated ASPM expression in eutopic endometrial tissues and derived primary stromal cells. Functional assays revealed that ASPM knockdown reduced endometrial stromal cell proliferation, invasion, and migration, whereas its overexpression enhanced these cellular processes. Transcriptome sequencing of ASPM-silenced stromal cells implicated cell cycle regulation in ASPM’s mechanism of action, with flow cytometry confirming G1 phase arrest following ASPM downregulation. ASPM modulation also altered Wnt/β-catenin signaling pathway activity, with rescue experiments demonstrating that Wnt/β-catenin inhibition counteracted ASPM overexpression effects.

**Conclusions:**

These results suggest ASPM promotes EMs progression via cell cycle regulation and Wnt/β-catenin signaling, offering novel insights into disease pathogenesis.

## 1. Introduction

Endometriosis (EMs) is a benign gynecological disorder involving functional endometrial tissue growth beyond the uterine cavity. Common clinical manifestations include pelvic pain, infertility, and adnexal masses, with frequent implantation sites encompassing the ovaries, uterosacral ligaments, and pelvic peritoneum.^[1]^ The pathogenesis of EMs remains incompletely understood, though existing evidence suggests links to dysregulated cell proliferation, invasion, and migration.^[2–4]^

Microarrays emerged from rapid progress in molecular biology techniques. Recent studies have leveraged bioinformatics approaches to analyze microarray data and identify differentially expressed genes (DEGs). Integrating multiple datasets overcomes the limitations of small sample sizes and high false-positive rates inherent in single microarray studies, producing more robust findings.

To elucidate the pathogenesis of EMs, we performed bioinformatics analyses that identified the abnormal spindle-like microcephaly-associated protein (ASPM) as a key regulator in EMs. ASPM, encoded by the primary microcephaly 5 (*MCPH5*) gene, regulates mitotic spindle formation and coordinates mitotic processes.^[5]^ Early research identified its role in controlling neuronal mitotic orientation and cytoplasmic division, which critically impacts cerebral cortex development.^[6]^ Later work demonstrated that ASPM enhances cell proliferation, invasion, and migration.^[7]^ The protein also functions in reproductive tissues, where Zhou et al^[8]^ observed increasing ASPM expression correlating with advanced endometrial cancer stages. Similarly, Alsiary et al^[9]^ detected strong cytoplasmic ASPM expression in ovarian cancer cells compared to moderate levels in normal ovarian tissue. Despite these findings, ASPM’s potential role in EMs remains unexplored.

Our previous pathway enrichment analysis revealed a significant association between DEGs and the cell cycle pathway. ASPM functions as a positive regulator of the Wnt signaling pathway.^[10]^ In the central nervous system, it mediates brain development through Wnt pathway modulation.^[11]^ Recent studies demonstrate that ASPM enhances colorectal cancer cell invasion and migration by promoting β-catenin nuclear translocation.^[12]^ The Wnt/β-catenin pathway contributes to EMs pathogenesis,^[13,14]^ with aberrant β-catenin activation increasing endometrial stromal cell (ESC) invasiveness.^[15]^

This study examined ASPM expression and its role in ESCs proliferation, invasion, and migration through cell cycle regulation and the Wnt/β-catenin pathway. These findings advance our understanding of EMs pathogenesis and offer initial insights into its underlying molecular mechanisms.

## 2. Methods

### 2.1. Microarray data analysis and identification of differentially expressed and hub genes

We retrieved 4 endometrial mRNA datasets (GSE11691, GSE25628, GSE7305, and E-MTAB-694) from the Gene Expression Omnibus and ArrayExpress databases, including 6 normal, 44 eutopic, and 45 ectopic endometrial tissue samples. The raw cell intensity files were processed using the “Affy” R package,^[16]^ with samples grouped by tissue type. Following robust multi‑array average normalization, we applied batch correction to the merged dataset. DEGs were filtered at *P* < .05 and |logFC| >1, with results displayed in volcano plots. Venn diagrams identified overlapping DEGs across datasets. Using the Search Tool for the Retrieval of Interacting Genes/Proteins database, we constructed a protein-protein interaction (PPI) network for shared DEGs, applying a combined score threshold of 0.9. Cytoscape visualized the PPI network, and the Cytohubba plugin detected hub genes based on a connectivity threshold of 10. Pathway enrichment analysis was conducted using the Kyoto Encyclopedia of Genes and Genomes. Following bioinformatics analysis, we identified a set of overlapping DEGs and constructed a PPI network. Cytohubba was then used to screen the ten hub genes with the highest connectivity, which were predominantly enriched in inflammation- and cell adhesion-related signaling pathways. These results establish a molecular basis for the pathogenesis of EMs and provide a foundation for the subsequent experimental validation of selected hub genes in clinical tissue samples.

### 2.2. Patients and tissues

We collected proliferative endometrial tissue samples from 40 patients admitted to our institution between December 2021 and April 2022, including 20 diagnosed with EMs and 20 controls. Controls were selected based on tubal or male factor infertility or laparoscopic confirmation of non-EMs conditions, while the EMs group comprised patients with laparoscopically confirmed stage III to IV ovarian EMs. Exclusion criteria included hormone-dependent diseases (e.g., uterine fibroids), recent hormonal therapy (within 3 months), pregnancy, a history of malignant tumors, or intraoperative detection of malignancy.

### 2.3. Culture of ESCs

Primary cells more accurately recapitulate disease states and individual variability than established cell lines, supporting their use in this study. Due to the limited availability of endometrial tissue, which was prioritized for routine clinical pathological examination, ESCs were successfully isolated from the proliferative phase endometrial tissues of only 6 patients with EMs and 8 unaffected controls. Immediately after collection, samples were placed in Dulbecco modified Eagle medium (DMEM)/F12 (Gibco) and transported to the laboratory. Tissues were washed 3 times with phosphate-buffered saline (PBS; Gibco) supplemented with penicillin–streptomycin to remove residual blood, then minced into 0.5 cm × 0.5 cm fragments and digested with trypsin (Gibco) for 1.5 to 2 hours. Following digestion, the cell suspension was sequentially filtered through 100-μm and 40-μm cell strainers to remove tissue debris and epithelial cells, yielding purified stromal cells. Isolated cells were maintained in DMEM/F12 supplemented with 10% fetal bovine serum (Gibco) and 1% penicillin–streptomycin under standard culture conditions (5% CO_2_, 37°C). Immunostaining for vimentin (1:1000, #MA5-16409, Invitrogen) and E-cadherin (1:200, #13-1700, Invitrogen) confirmed that stromal cell purity exceeded 95%, making them suitable for downstream applications.

### 2.4. Total RNA extraction and reverse transcription quantitative polymerase chain reaction

Total RNA was isolated from endometrial tissues and ESCs with TRIzol reagent (Invitrogen) following the manufacturer’s protocol. We reverse-transcribed 1 μg of total RNA into cDNA using a dsDNase-treated first-strand cDNA synthesis kit (Thermo Fisher Scientific). Reverse transcription quantitative polymerase chain reaction (RT-qPCR) analysis was conducted on an ABI7500 system with SYBR Premix (Applied Biosystems), with GAPDH serving as the endogenous control. All reactions were performed in triplicate. The primer sequences were: ASPM forward 5′-TGCAGTGGGTGAACATGAAAA-3′, reverse 5′-CGAAGAGGGTGTTACCTCGTTT-3′; GAPDH forward 5′-ATGGAAATCCCATCACCATCTT-3′, reverse 5′-CGCCCCACTTGATTTTGG-3′.

### 2.5. Protein extraction and western blotting

ESCs lysates were prepared using radioimmunoprecipitation assay buffer (Beyotime) containing 1× protease phosphatase inhibitor (Thermo Fisher Scientific), with protein concentrations determined by bicinchoninic acid assay (Beyotime). Proteins were resolved on 7.5% to 12.5% sodium dodecyl sulfate polyacrylamide gel electrophoresis gels based on molecular weight and electrotransferred to polyvinylidene fluoride membranes. After blocking, membranes were probed overnight at 4°C with primary antibodies targeting ASPM (1:500, 26223-1-AP, Proteintech), β-catenin (1:2000, #MA1-301, Invitrogen), CyclinD1 (1:1000, #MA5-14512, Invitrogen), c-myc (1:1000, #13-2500, Invitrogen), and GAPDH (1:5000, 10494-1-AP, Proteintech). Following 3 washes with Tris-buffered saline with Tween 20, membranes were incubated with peroxidase-conjugated secondary antibodies (1:10000, C31460100/C31430100, Invitrogen) for 1 hour at room temperature. Protein signals were visualized using a ChemiDoc system (Bio-Rad) with enhanced chemiluminescence substrate (Epizyme).

### 2.6. Immunohistochemistry

Endometrial tissues and ESCs were fixed in 4% paraformaldehyde for 20 minutes, embedded in paraffin, and sectioned. After dewaxing with xylene and rehydration through an ethanol gradient, the sections were incubated at 60°C for 30 minutes. Antigen retrieval was performed by microwaving the sections in citrate buffer (pH 6.0) for 4 to 6 minutes, followed by passive cooling and PBS rinses. Endogenous peroxidase activity was quenched by treating the sections with 3% hydrogen peroxide at room temperature for 15 minutes. Following blocking with goat serum, the sections were incubated overnight at 4°C with primary antibodies targeting ASPM (1:500, 26223-1-AP, Proteintech), vimentin (1:1000, #MA5-16409, Invitrogen), and E-cadherin (1:2000, #13-1700, Invitrogen). After PBS washes, peroxidase-conjugated secondary antibodies were applied for 30 minutes at room temperature. The sections were developed with diaminobenzidine, counterstained with hematoxylin, dehydrated, and mounted for microscopic evaluation using H-scores.

### 2.7. Cell transfection

GenePharma synthesized both the ASPM-targeting siRNA and the negative control siRNA (si-NC). The ASPM-specific siRNA (si-ASPM) contained the forward sequence 5′-GGAAUCAACAAUUGCACAUTT-3′ and the reverse sequence 5′-AUGUGCAAUUGUUGAUUCCTT-3′, while the si-NC sequences were 5′-UUCUCCGAACGUGUCACGUTT-3′ (forward) and 5′-ACGUGACACGUUCGGAGAATT-3′ (reverse). ESCs (1 × 10^6^) were seeded in 6-well plates 24 hours before transfection. Transfection was performed using 30 pmol of either si-ASPM or si-NC with Lipofectamine RNAiMAX (Invitrogen). For plasmid transfection, the GV230-ASPM vector, constructed by GeneChem, and the control plasmid (2.5 μg each) were delivered using Lipofectamine 3000 (Invitrogen). Cells were harvested 48 hours post-transfection for subsequent RT-qPCR and western blotting.

### 2.8. Cell counting kit-8 assay

ESCs subjected to various treatments were plated in 96-well plates. After adding cell counting kit-8 (CCK-8) solution (Merck KGaA), cells were incubated for 3 hours at 37°C under 5% CO_2_. Absorbance measurements at 450 nm were recorded at 0, 24, 48, and 72 hours using a Thermo Fisher Scientific microplate reader.

### 2.9. Transwell assay

ESCs treated under various conditions were seeded into the upper transwell chamber at 5000 cells per well. The lower chamber received 700 μL of DMEM/F12 supplemented with 20% fetal bovine serum. Following a 48-hour incubation, non-migrated cells on the membrane’s upper surface were removed, while migrated cells beneath the membrane were fixed with 4% paraformaldehyde and stained with crystal violet. Three randomly selected fields per chamber were imaged at 200× magnification using light microscopy, and the average cell count per field was determined. For invasion assays, the upper chamber was precoated with 60 μL of Matrigel (NovaMedica) diluted 1:8, followed by identical procedures as the migration assay.

### 2.10. Wound-healing assay

Forty-eight hours post-intervention, ESCs were plated in 6-well plates using distinct protocols. After reaching 90% confluence, 3 parallel scratches were created in each well with 10-μL pipette tips, and scratch widths were measured immediately. Cells were maintained in serum-free DMEM/F12, with scratch distances quantified at 24- and 72-hour intervals.

### 2.11. Cell cycle assay

Following treatment, ESCs were harvested, washed with PBS, and incubated with 1 mL of DNA staining solution and 10 μL of permeabilization solution (MultiSciences). After 30 minutes of dark incubation at room temperature, samples were analyzed on a BD FACSCanto II flow cytometer.

### 2.12. Whole-genome expression profile analysis

RNA was extracted from cells transfected with either si-NC or si-ASPM siRNA, with 3 biological replicates per group. BGI Technology Co., Ltd. conducted the RNA sequencing. Clean reads were aligned to the reference genome using Bowtie2 (v2.3.4.3; Johns Hopkins University),^[17]^ and gene expression quantification was performed with RSEM (v1.3.1; University of Wisconsin-Madison).^[18]^ Differential expression analysis, implemented in DESeq2 (v1.4.5; EMBL/Harvard),^[19]^ identified significant genes at a threshold of *Q* ≤ 0.05 or false discovery rate ≤0.001.

### 2.13. Co-immunoprecipitation

A total of 10 μg of anti-ASPM antibody was incubated with 20 μL of coupling resin at room temperature for 2 hours. The mixture was then stored at 4°C according to the co-immunoprecipitation kit manufacturer’s protocol (Thermo Scientific). ESCs were lysed on ice in IP lysis/wash buffer for 5 minutes, and the supernatant was collected. After incubating 1 mg of cell lysate with 80 μL of control agarose resin at 4°C for 1 hour, we collected the flow-through solution. The bait protein was combined with the target protein and incubated overnight at 4°C. Immune complexes were eluted from the resin using 60 μL of elution buffer. These complexes were then analyzed by sodium dodecyl sulfate polyacrylamide gel electrophoresis and western blotting.

### 2.14. Statistical analysis

Statistical analyses were performed with SPSS 26.0 (SPSS Inc.), GraphPad Prism 8, and ImageJ 1.53k (NIH). Results are expressed as mean ± SD. Intergroup comparisons used two-tailed Student’s *t* tests, with statistical significance set at *P* < .05.

## 3. Results

### 3.1. Identification of the hub gene ASPM

**Figure 1.**
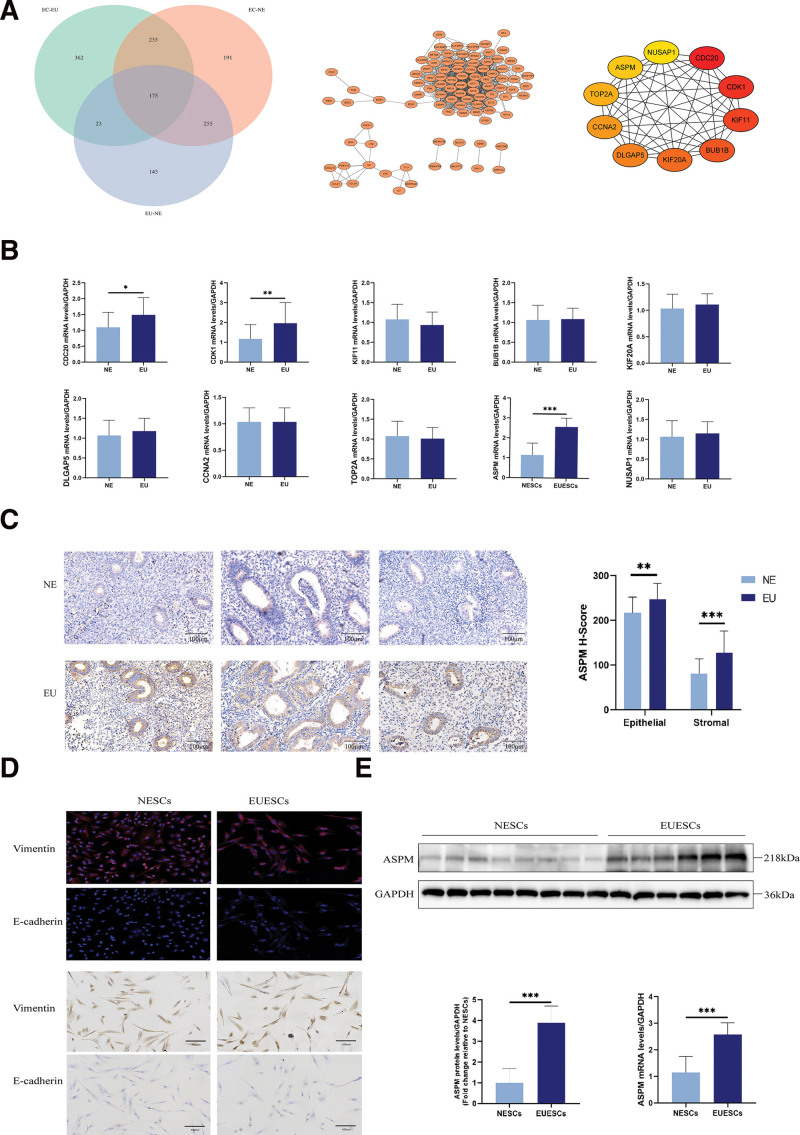
The screening and validation of ASPM. (A) Identification and enrichment analysis of DEGs. (B) Verification of hub genes by RT-qPCR. (C) Detection of ASPM expression by IHC. (D) The cultivation and identification of ESCs. (E) RT-qPCR and western blot analysis of mRNA and protein levels of ASPM in ESCs. *P < .05, **P < .01, ***P < .001. ASPM = abnormal spindle-like microcephaly-associated protein, DEGs = differentially expressed genes, ESCs = endometrial stromal cells, IHC = immunohistochemistry, RT-qPCR = reverse transcription quantitative polymerase chain reaction.

Four mRNA datasets for EMs (GSE11691, GSE25628, GSE7305, and E-MTAB-694) were retrieved from Gene Expression Omnibus and ArrayExpress, including 6 normal endometrial samples, 44 eutopic endometrial samples, and 45 ectopic endometrial samples (Table S1). Comparative analysis identified 175 overlapping DEGs across these datasets. The Search Tool for the Retrieval of Interacting Genes/Proteins database generated a PPI network for these DEGs, which Cytoscape subsequently visualized. CytoHubba analysis revealed *CDC20*, *CDK1*, *KIF11*, *BUB1B*, *KIF20A*, *DLGAP5*, *CCNA2*, *TOP2A*, *ASPM*, and *NUSAP1* as the most interconnected hub genes (Fig. 1A).

### 3.2. Demographics

Endometrial tissues in the proliferative phase were obtained from 20 patients with EMs and 20 unaffected controls. Table 1 presents the demographic characteristics of all participants. The 2 groups showed no statistically significant differences.

**Table 1:** Demographic and baseline date.

Parameters	Non-EMs (n = 20)	EMs (n = 20)	*P* value
Age (yr)	29.35 ± 3.67	31.10 ± 3.83	.149*
BMI (kg/m^2^)	21.22 ± 1.48	20.70 ± 1.77	.392*
Gravidity (times)	2.5 (2–3)	2 (2–3.75)	.862†
Parity (times)	1 (1–1)	1 (1–2)	.231†
Abortion (times)	2 (1–2)	1.5 (1–3)	.640†
Menarche (yr)	13 (13–14)	13 (12–14.75)	.512†
Cycle length			.603‡
≤24 d	1	2	
25–29 d	6	10	
30–34 d	13	8	
Length of menstruation			.770‡
≤4 d	7	5	
5–6 d	10	11	
≥7 d	3	4	

BMI = body mass index, EMs = endometriosis.

* *t* test.

† Mann–Whitney *U* test.

‡ Fisher exact test.

### 3.3. High ASPM expression was detected in eutopic endometrial tissues and their derived ESCs

We validated 10 hub genes via RT-qPCR in endometrial tissues from EMs patients and controls, identifying ASPM as the most DEG (Fig. 1B). Subsequent analyses focused on ASPM expression patterns. Immunohistochemistry revealed elevated ASPM levels in EMs endometrium compared with control tissues (Fig. 1C). Primary ESCs were isolated from ectopic endometrium of 6 EMs patients and eutopic endometrium of 8 controls. Immunohistochemistry and immunofluorescence staining for vimentin and E-cadherin confirmed >90% cell purity (Fig. 1D). ESCs from EMs patients exhibited significantly higher ASPM mRNA (*P* < .001) and protein (*P* < .001) expression than controls (Fig. 1E).

### 3.4. Knockdown of ASPM suppressed the proliferation, invasion, and migration of ESCs

**Figure 2.**
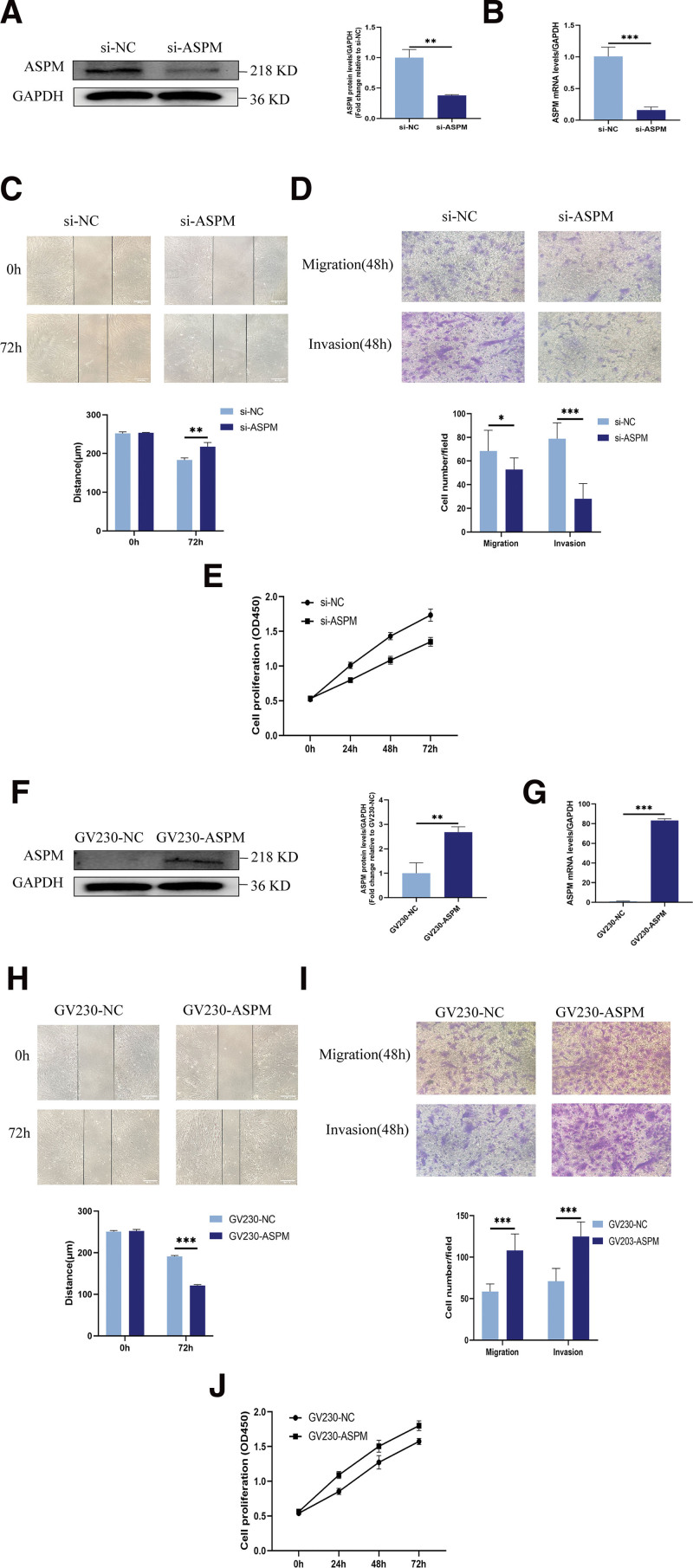
Knockdown and overexpression of ASPM affected the proliferation, invasion, and migration of ESCs. (A and B) Western blotting and RT-qPCR analysis of ASPM protein and mRNA levels after transfection of ESCs with si-NC or si-ASPM. (C and D) Wound-healing and transwell assays to examine the migration and invasion ability of ESCs. (E) CCK-8 assay to examine the proliferation ability of ESCs. (F and G) Western blotting and RT-qPCR analysis of ASPM protein and mRNA levels after transfection of ESCs with GV230-NC or GV230-ASPM. (H and I) Wound-healing and transwell assays to examine the migration and invasion ability of ESCs. (J) CCK-8 assay to examine the proliferation ability of ESCs. *P < .05, **P < .01, ***P < .001. ASPM = abnormal spindle-like microcephaly-associated protein, CCK-8 = cell counting kit-8, ESCs = endometrial stromal cells, RT-qPCR = reverse transcription quantitative polymerase chain reaction.

To investigate ASPM’s function in EMs, we transfected ESCs from EMs patients with ASPM-targeting siRNA. ASPM mRNA (*P* < .001) and protein (*P* < .01) expression significantly decreased 48 hours post-transfection (Fig. 2A, B). Wound-healing (*P* < .01) and transwell (*P* < .05) assays demonstrated reduced migratory and invasive capacity in si-ASPM-treated ESCs relative to si-NC controls (Fig. 2C, D). CCK-8 assays revealed impaired proliferation in ESCs following ASPM knockdown (Fig. 2E).

### 3.5. ASPM overexpression enhanced the proliferation, invasion, and migration of ESCs

GV230-mediated ASPM overexpression in ESCs from non-EMs patients significantly elevated ASPM mRNA (*P* < .001) and protein (*P* < .01) levels 48 hours post-transfection (Fig. 2F, G). Wound-healing (*P* < .001) and transwell (*P* < .05) assays revealed greater migration and invasion capacities in GV230-ASPM-transfected ESCs than in GV230-NC controls (Fig. 2H, I). CCK-8 assays further confirmed increased proliferation in GV230-ASPM-transfected ESCs relative to GV230-NC-transfected cells (Fig. 2J).

### 3.6. ASPM modulates ESC proliferation, invasion, and migration through cell cycle regulation and Wnt/β-catenin signaling

**Figure 3.**
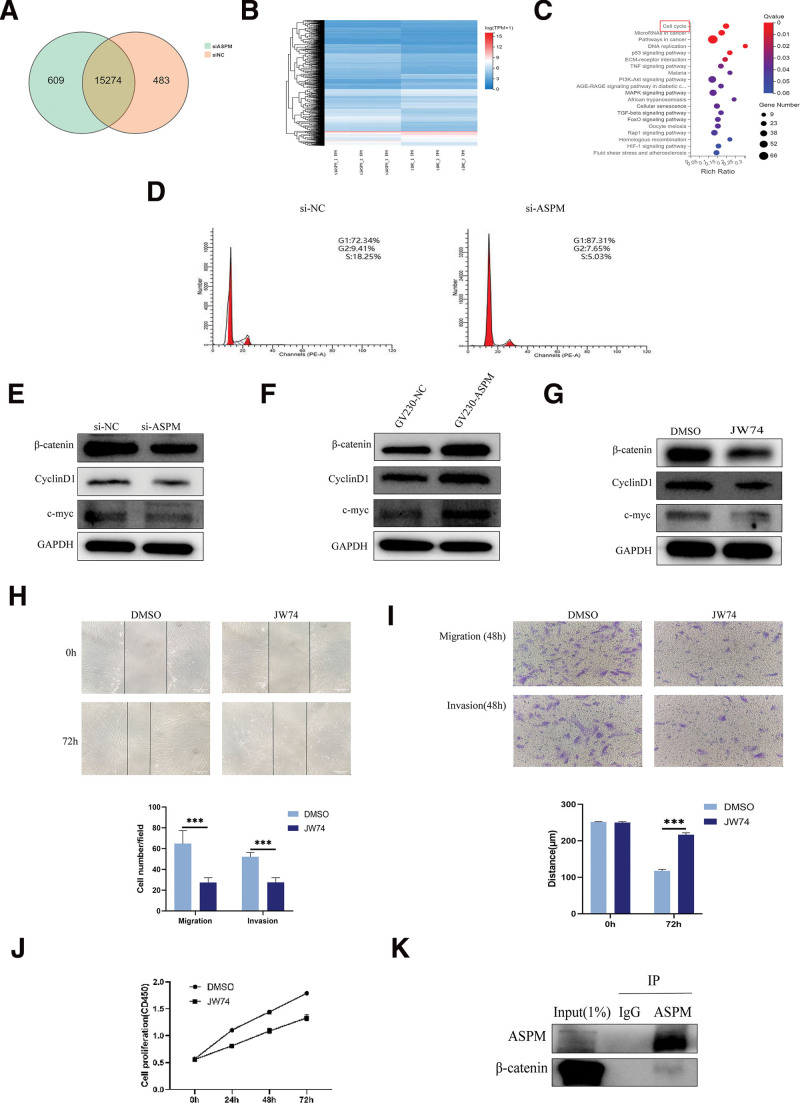
ASPM regulated the proliferation, invasion, migration of ESCs via the cell cycle and Wnt/β-catenin pathway. (A and B) Wayne diagram and heat map of DEGs between the si-ASPM group and the si-NC group. (C) KEGG enrichment analysis of DEGs. (D) The cell cycle distribution of ESCs with ASPM knockdown was determined by flow cytometry. (E–G) Western blotting analysis of β-catenin, cyclinD1, and c-myc protein levels. (H and I) Wound-healing and transwell assays to examine the migration and invasion ability of ESCs. (J) CCK-8 assay to examine the proliferation ability of ESCs. (K) Interaction of ASPM and β-catenin was examined by Co-IP in ESCs. ASPM = abnormal spindle-like microcephaly-associated protein, CCK-8 = cell counting kit-8, Co-IP = co-immunoprecipitation,DEGs = differentially expressed genes, ESCs = endometrial stromal cells, KEGG = Kyoto Encyclopedia of Genes and Genomes.

Transcriptome analysis comparing ASPM-knockdown ESCs with controls identified 15,274 DEGs (Fig. 3A, B). Kyoto Encyclopedia of Genes and Genomes analysis highlighted cell cycle pathway involvement (Fig. 3C). Cell cycle assays confirmed G1 phase arrest following ASPM knockdown (Fig. 3D). Given prior evidence linking ASPM to Wnt/β-catenin signaling,^[11,20]^ we assessed pathway activity after transfecting ESCs with si-ASPM or GV230-ASPM. si-ASPM reduced β-catenin, cyclinD1, and c-myc expression, whereas GV230-ASPM elevated these protein levels (Fig. 3E, F). Rescue experiments using the Wnt/β-catenin inhibitor JW74 further supported this regulatory mechanism. JW74-treated ESCs exhibited lower β-catenin, cyclinD1, and c-myc expression than DMSO controls after 48 hours (Fig. 3G). Wound-healing (*P* < .001; Fig. 3H) and transwell assays (Fig. 3I) revealed impaired ESC migration and invasion in the JW74 group, while CCK-8 assays confirmed reduced proliferation (Fig. 3J). Co-immunoprecipitation experiments demonstrated physical interaction between ASPM and β-catenin (Fig. 3K), corroborating their functional association.

## 4. Discussion

EMs is a common gynecological disorder that shares key features with malignant tumors, with pathogenesis closely tied to the proliferation, invasion, and migration of ESCs. The disease involves complex molecular interactions and signaling pathway dysregulation. Identifying new regulatory targets could advance therapeutic development. Our findings demonstrate that ASPM functions as a central regulator in EMs, showing elevated expression in both eutopic endometrial tissues and derived ESCs. ASPM mediates ESC proliferation, invasion, and migration by influencing cell cycle progression and Wnt/β-catenin signaling.

ASPM, a centrosomal protein implicated in embryonic development, cell division, and proliferation,^[21,22]^ has been identified as a potential biomarker for multiple malignancies. Originally linked to microcephaly, ASPM expression levels correlate with poor prognosis across several cancers. Deng et al^[23]^ demonstrated that elevated ASPM expression predicts adverse outcomes and increased immune cell infiltration in renal clear cell carcinoma and hepatocellular carcinoma. Tang et al^[24]^ reported similar prognostic significance in breast cancer, while Zhou et al^[8]^ established this association in endometrial cancer. Earlier work^[8,9]^ further confirmed ASPM’s relationship with advanced endometrial and high-grade ovarian cancers. Despite these findings, ASPM’s role in EMs remains unexplored. Here, we present the first evidence of ASPM’s elevated expression in EMs and propose its potential molecular mechanisms in this condition. These observations prompted our investigation into ASPM’s specific functions in EMs.

EMs exhibits malignant tumor-like characteristics such as uncontrolled cell proliferation, migration, and invasion.^[25]^ Early evidence for ESC invasiveness emerged from studies examining the cellular composition of EM implants.^[26]^ Comparative analyses demonstrated that ESCs from EM patients proliferate, migrate, and stimulate angiogenesis more aggressively than those from healthy controls. A detailed investigation of ESCs isolated from peritoneal, ovarian, and deeply infiltrating endometrial lesions revealed altered integrin expression patterns that enhance cellular adhesion and proliferation in EM patients.^[27]^ Our functional studies of ASPM showed that its knockdown suppressed ESC proliferation, invasion, and migration, while its overexpression exacerbated these phenotypes, clarifying ASPM’s involvement in EM pathogenesis.

The Wnt signaling pathway serves as a critical regulator of growth processes spanning oncogenesis to developmental biology.^[28]^ β-catenin mediates Wnt pathway activation, which occurs during both embryo implantation and tumor development.^[29,30]^ Aberrant Wnt/β-catenin signaling has been implicated in EM pathogenesis,^[31]^ where it facilitates epithelial-mesenchymal transition by disrupting cell polarity and promoting motility.^[32]^ This pathway is particularly important for ESC invasion in EM.^[13,33]^ As a Wnt pathway activator involved in meiotic regulation,^[13,33]^ ASPM positively modulates Wnt/β-catenin signaling through β-catenin interaction, providing mechanistic insight into EM progression. ASPM-mediated cell cycle control at the G1 checkpoint^[34]^ and its influence on Wnt pathway activity^[35]^ were confirmed by our observation that ASPM knockdown triggers G1-phase arrest in stromal cells, aligning with established mechanisms.

This study has several limitations. Ethical constraints limited the control group to patients with tubal factor infertility without severe complications. Although we uncovered preliminary molecular mechanisms involving ASPM, the study did not explore potential upstream regulators or downstream targets. Future work should elucidate ASPM’s precise molecular function in EMs and its interaction with β-catenin.

In summary, our findings demonstrate that ASPM regulates embryonic stem cell proliferation, invasion, and migration by modulating the cell cycle and Wnt/β-catenin signaling. This effect may occur through direct interaction with β-catenin.

## Author contributions

**Funding acquisition:** Xin Liu, Fengque Zheng.

**Methodology:** Xin Liu, Rongyan Qin.

**Writing – review & editing:** Rongyan Qin, Fengque Zheng.

**Conceptualization:** Fengque Zheng.

**Writing – original draft:** Fengque Zheng.


