# Supplementary material for: ASPM promotes the progression of ovarian endometriosis by modulating the cell cycle and activating the Wnt/β-catenin signaling pathway
Source: Medicine (Baltimore). 2026 May 15;105(20):e48765. doi: 10.1097/MD.0000000000048765 (PMC13183157; doi:10.1097/MD.0000000000048765)
Supplement: Supplementary file 1 [file medi-105-e48765-s001.docx]

Table S1 Basic information about the microarray datasets of three different endometrium

| Datasets | Samples | | | Platform | References |
| --- | --- | --- | --- | --- | --- |
|  | NE | EU | EC |  |  |
| GSE11691 | - | 9 | 9 | GPL96 | Hull ML et al. (2008) |
| GSE25628 | 6 | 8 | 8 | GPL571 | Crispi et al. (2013) |
| GSE7305 | - | 10 | 10 | GPL570 | Hever et al. (2007) |
| E-MTAB-694 | - | 17 | 18 | GPL570 | Sohler et al. (2013) |
